# Evaluation of the implementation of centralized waiting lists for patients without a family physician and their effects across the province of Quebec

**DOI:** 10.1186/s13012-014-0117-9

**Published:** 2014-09-04

**Authors:** Mylaine Breton, Astrid Brousselle, Antoine Boivin, Christine Loignon, Nassera Touati, Carl-Ardy Dubois, Kareen Nour, Djamal Berbiche, Danièle Roberge

**Affiliations:** Charles-LeMoyne Hospital Research Centre, Greenfield Park, J4K 0A8 QC Canada; Faculty of Medicine and Health Sciences, Université de Sherbrooke-Campus Longueuil, Longueuil, J4K 0A8 QC Canada; École Nationale d'Administration Publique, Montreal, G1K 9E5 QC Canada; Faculty of Nursing, University of Montreal, Montreal, H3C 3J7 QC Canada; Montérégie Regional Department of Public Health, Longueil, J4K 2M3 QC Canada

**Keywords:** Primary healthcare, Access, Continuity, Implementation study

## Abstract

**Background:**

Most national and provincial commissions on healthcare services in Canada over the past decade have recommended that primary care services be strengthened in order to guarantee each citizen access to a family physician. Despite these recommendations, finding a family physician continues to be problematic. The issue of enrolment with a family physician is worrying in Canada, where nearly 21% of the country's population reported not having a family physician in the last Commonwealth Fund survey.

To respond to this important need, centralized waiting lists have been implemented in four Canadian provinces to help `orphan,' or unaffiliated, patients find a family physician. These organizational mechanisms are intended to better coordinate the demand for and supply of family physicians. The objectives of this study are: to assess the effects of centralized waiting lists for orphan patients (GACOs) implemented in the province of Quebec and to explain the variation among their effects by analyzing factors influencing implementation process.

**Methods:**

This study is based on two complementary and sequential research strategies. The first (objective 1) is a quantitative longitudinal design to assess the effects of all the GACOs (n = 93) in Quebec using clinical-administrative data. The second (objective 2) involves using four case studies to explain variations in effects through in-depth analysis of the various factors contributing to the observed effects. The primary source of data will be key actors involved in the GACOs. We expect to conduct around 40 semi-structured interviews.

**Discussion:**

This will be the first study in Canada to evaluate the implementation of this innovation. It will provide an exhaustive picture of the effects of GACO implementation in Quebec and to assess their potential for generalization elsewhere in Canada. At the theoretical level, this study will produce new knowledge on the factors having the greatest influence on the implementation of primary care innovations in professional environments.

**Electronic supplementary material:**

The online version of this article (doi:10.1186/s13012-014-0117-9) contains supplementary material, which is available to authorized users.

## Background

### Need for improved access to family physicians

Several Canadian and provincial commissions on healthcare have recommended that primary care services be reinforced to guarantee every citizen access to a family physician who would, on one hand, be the primary source of most care, and on the other hand, work in a clinical setting that provides primary care that is accessible, continuous, comprehensive, and well coordinated with other levels of care [1]-[6]. These recommendations are based on strong evidence about the benefits of good patient management in primary care [7]-[9]. The literature shows that vulnerable patients are the ones who benefit most from good quality primary care [10],[11].

The benefits associated with having a family doctor, in terms of quality of care (*e.g.*, preventive care activities) and care outcomes (*e.g.*, patient satisfaction, compliance with treatment, better use of services) have been extensively documented [12]-[18]. The benefits are particularly clear with regard to routine care and follow-up, and somewhat less so for needs for immediate care for a minor problem [19].

Patients with chronic illnesses require regular follow-up with a primary care professional to ensure good management of their condition [20]. According to several authors, better primary care management of chronic illnesses improves health status, which decreases the probability of complications or clinical deterioration that could result in an emergency room visit or hospitalization [21]-[23]. Thus, the literature shows that patients with chronic illness who have no family physician are at higher risk of having poor health status [23] and of using the emergency room or being hospitalized for foreseeable and avoidable health conditions (*e.g.*, patient with decompensated diabetes). According to a recent Canadian study, people with a chronic health problem and no family physician were 1.2 times more likely to use emergency services and 1.3 times more likely to be admitted to hospital (non-elective admission) [24]. Several countries, in fact, use rates of hospitalization for certain conditions as indicators of limited access to primary care services [21]. Emergency rooms are considered to be an inadequate and non-optimal substitute for primary care for ongoing health problems and chronic illness management [23].

Affiliation with a family physician is also considered to be a prerequisite to accessibility [13]-[15]. Accessibility refers to the ease or difficulty with which a person is able to establish or maintain contact with a care provider [15]-[17]. Accessibility and continuity of care are complementary. Accessibility is a prerequisite for relational continuity, which in turn facilitates accessibility to one's family physician or to another physician practising in the same medical clinic [25]. Continuity refers to the degree to which the healthcare services provided are carried out without interruption over time, within one or more episodes of care [26]-[30].

Several authors also assert that establishing a therapeutic patient-physician relationship contributes to care continuity [31],[32]. Numerous studies have shown the positive effects associated with care continuity, including greater satisfaction on the part of both patients and physicians, improved compliance with treatments, reduced emergency room usage, and fewer hospitalizations [33]. As well, it is especially important for patients to be able to count on a primary care provider who knows their personal situation and is the primary person in charge of their care, an attribute which we refer to as relational continuity [8]. Relational continuity is defined by a therapeutic relationship between a patient and one or more care providers, over a span of time and for various health services, which results in providers' accumulated knowledge about the patient as well as services that are consistent with the patient's needs [8]. Several studies suggest that this relational aspect is a very high priority for patients [8],[34]-[38]. Likewise, the Institute of Medicine considers a sustained patient-physician relationship to be a core element of primary care [39].

Despite these many recommendations, the issue of affiliation with a family physician remains a concern for Canadians, as nearly 21% of the country's population reported not having a family physician in the last Commonwealth Fund survey [40]. This situation is even more pronounced in Quebec, where nearly 29% of the population reported not having a family physician, the worst score in Canada [40]. In addition, of the residents of Quebec without a family physician, 60% reported that they had been unable to find a physician when they needed one [18]. Not having a family physician is especially critical for patients with the greatest needs (*e.g.*, those who perceive themselves to be in poor health, or have one or more chronic illnesses, or have been hospitalized or undergone major surgery). For these patients presenting the greatest needs, Quebec again is in last place among the Canadian provinces in terms of patients without a family physician, as nearly 16% of these patients fall in that category [41]. This state of affairs is not unique, as vulnerable clienteles are known to experience significant difficulty in accessing primary care (inverse care law) [11],[42],[43] and receive the fewest preventive and screening services in primary care [9]. The considerable number of patients without a family physician in Canada is therefore a major issue [31],[44].

### Research opportunity: Innovation implemented to help patients find a family physician

In response to this problem of affiliation with a family physician, especially for more vulnerable clienteles, several Canadian provinces have set up centralized waiting lists to better coordinate primary care medical resources, notably in Ontario (Health Care Connect), British Columbia (A GP for Me), New Brunswick (Health Connect NB), and Quebec (GACO: Guichet d'accès aux clienteles orphelines - waiting list for `orphan' clienteles). These waiting lists are considered to be organizational innovations to improve access to family physicians and to foster continuity of care. They are used to centralize requests for family physicians in a given territory and to match orphan patients with a family physician, based on a priority scale and on the availability of primary care physicians. To our knowledge, no study has yet examined the implementation and outcomes of these new and complex organizational models, apart from one exploratory study we conducted in one region of Quebec [45].

In a context in which health resources are limited and the population's expectations are not met, it is important to assess whether these centralized waiting lists are actually effective in meeting the targeted objectives in terms of primary care accessibility and continuity. For example, in the most recent fiscal year in Quebec (2012-2013), family physicians received nearly $38 million in bonuses for enrolling orphan patients through GACOs, not counting the other resources allocated to GACO operations, such as the nurses, secretaries, and physician coordinators working there.

### Study setting

Quebec is a province of over eight million residents with a tax-based system providing universal access to medical services. Healthcare organizations, such as local community health centres (CLSC) and hospitals, receive block funding from the Ministry of Health and Social Services (MSSS), and physicians are remunerated predominantly on a fee-for-service basis. While nearly all family physicians provide medical services reimbursed by the public Medicare program, most primary healthcare practices are private rather than public enterprises.

In Quebec, 93 GACOs were set up in 2008. Each of these waiting lists is overseen by a Health and Social Services Centre (CSSS), which is responsible for the population of a given territory. The aim of this policy is to facilitate the local population's access to family physicians based on a clinical priority scale and on the availability of medical personnel in that territory.

The GACOs are managed by a secretary and a nurse, in collaboration with a local physician coordinator. Requests for registration in a GACO may come directly from patients or from referring health professionals (nurses, social workers, physicians). Once registered on a centralized waiting list, patients are assessed by the nurse, who determines their priority code according to the urgency and complexity of their healthcare needs. Patients are then enrolled with a family physician based on medical staff availability and the fields of practice of the physicians registered with the GACO, taking into consideration as much as possible the established priorities.

A physician who accepts an orphan patient through the GACO receives a financial bonus upon the patient's first visit. This financial incentive was implemented to encourage physicians' participation in the GACOs. The amount of the incentive depends on whether the patient has been designated as vulnerable. Patients are considered vulnerable if they present one of the 19 vulnerability codes defined by Quebec's health insurance board (RAMQ) based on the presence of medical diagnoses (*e.g.*, diabetes, chronic obstructive pulmonary disease, mental health disorder). This vulnerability code is different from the priority code determined by the nurse, but it influences that code, which establishes the patient's medical condition.

### The general objective of this study is to assess the impacts of the GACOs on service accessibility and continuity and to explain variations in these impacts

The specific objectives are:

1. To assess the impacts of GACOs on accessibility, continuity, and emergency room use;
2. To explain variations in the impacts by analyzing the characteristics of the GACOs and other factors influencing their implementation.

### Conceptual model based on implementation of a healthcare system innovation

**Figure 1:**
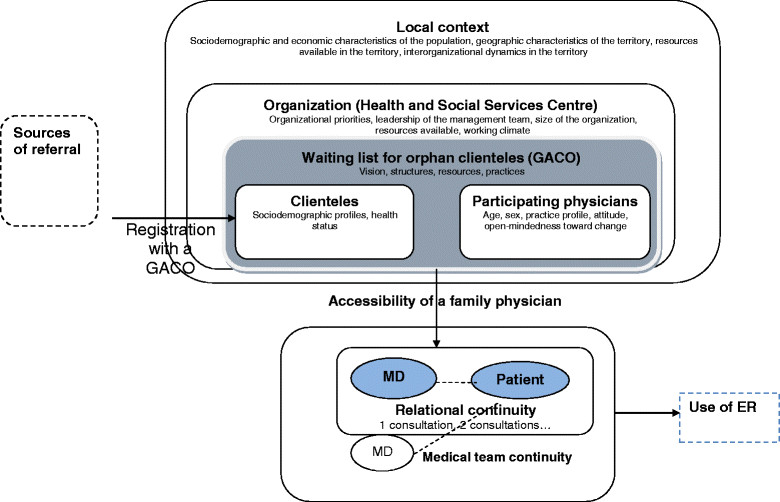
**Conceptual model.**

Analyzing the implementation of interventions such as those associated with waiting lists involves studying how, in a particular context, they produce the observed effects [46]. This type of analysis examines variations in context and in the implementation of the intervention in relation to the outcomes observed. We will base our study on a health innovations analysis model proposed by Chaudoir *et al.*[47] that focuses on carrying out a systematic review of the topic [47]. This model suggests paying attention to four types of factors: characteristics of the professionals involved in implementing the intervention; characteristics of the clienteles affected by the intervention; characteristics of the organization in which the intervention is implemented; and characteristics of the context. These groups of factors are described in the following paragraphs. Figure 1 shows the conceptual model on which this research is based.

The characteristics of the professionals involved in implementing the intervention refer to individual characteristics such as age, practice profile, and attitude or open-mindedness toward change [48]. Certain characteristics of clienteles targeted by the intervention also appear to influence the form of the intervention implemented, as it is adapted in response to the clienteles' characteristics. These characteristics include their sociodemographic and socioeconomic profiles as well as health status [49].

The characteristics of organizations can also influence the implementation of innovations, notably organizational priorities, the leadership of the management team, the size of the organization, the resources available, and the working climate [50]-[53]. Several studies have shown that one of the most important success factors for an intervention's implementation is having designated decision-makers and a governance structure to implement the intervention [50],[54], as well as protected funding for its operations. Likewise, the organization's values guide its various priorities, which in turn influence the allocation of resources to the intervention [55],[56].

Several characteristics of the local context can influence an intervention's implementation, including, in particular, the sociodemographic and socioeconomic characteristics of the population (*e.g.*, proportion of elderly, proportion of low-income persons), the geographic characteristics of the territory (*e.g.*, rural, urban), the resources available in the territory (*e.g.*, number of medical clinics, presence of a hospital), and the interorganizational dynamics (*e.g.*, collaborations among the medical clinics, and between the clinics and the CSSS) [52]. Studies have shown different effects for the same primary care models depending on the urban-rural geographic context [57],[58]. Another study comparing primary care organizational models showed that the geographic context had a determining influence on medical practice [59]. Finally, there are contextual factors at the macroscopic level that also influence the implementation of innovations, such as funding models and healthcare system regulatory models, as well as the number and diversity of healthcare resources [60].

## Methods

### Research design

This study is based on two complementary and sequential research strategies [61]. The first strategy (objective 1) will use a longitudinal design to assess the effects of all the GACOs (n = 93) in Quebec. For this purpose, we will use two clinical-administrative databases. The second strategy (objective 2) will use the case study approach (multiple case study) to explain the variations in the effects through in-depth analysis of the different factors contributing to the observed effects. Case study is particularly indicated for gaining a deeper understanding of implementation processes in relation to effects [46].

### Objective 1: Assess the impacts of GACOs on accessibility, continuity, and emergency room use

The main objective of the GACOs is to match one patient with one family physician so that the patient will be followed regularly by the family physician and will consult that physician when there is a need. To assess the effects, we will focus on: the family physician's accessibility; continuity of care; and use of emergency care. Table 1 summarizes the effects analyzed, the variables used, the definitions and reference periods, as well as the data sources. Achieving objective 1 will allow us to describe the effects of each GACO in Quebec and trace the evolution of the GACOs, and will facilitate the selection of cases for the qualitative component of the study.

**Table 1:** **Summary of quantitative variables analyzed, variables used, definitions, reference periods, and data sources**

Effects	Variables	Definition and reference period	Data sources
Accessibility	Patients enrolled with a family physician through the GACO	Number of patients enrolled with a family physician through the GACOs per 10,000 population per year	SIGACO, 2011 census data
	Vulnerable patients enrolled with a family physician through the GACO	Number of vulnerable patients enrolled with a family physician through the GACOs per 10,000 population per year	SIGACO, 2011 census data
Continuity	Visits to the family physician with whom the patient was enrolled through the GACO	Mean annual number of visits to the family physician with whom the patient was enrolled through the GACO	RAMQ
	Attendance rate	Annual rate of primary care visits with the physician with whom the patient was enrolled through the GACO compared with other primary care visits to a family physician, including in the ER, since the patient was enrolled with the family physician through the GACO	RAMQ
	Medical team continuity	Annual rate of visits to the medical clinic where the physician with whom the patient is enrolled practises, compared with visits to other clinics, including the ER	RAMQ
ER use before/after GACO	ER visits	Mean annual number of visits to the ER both before and after enrollment with a family physician through the GACO	RAMQ
	Rate of ER visits	Annual rate of visits to the ER compared with other primary care visits	RAMQ
	Visits to the ER to see the family physician with whom the patient was enrolled through the GACO	Annual rate of visits in the ER with the family physician with whom the patient was enrolled through the GACO (exploratory), compared with other primary care visits since the patient was enrolled with that family physician through the GACO	RAMQ

### Variables to be studied

#### Accessibility to a family physician

The creation of GACOs targeted two objectives related to family physician accessibility: to increase the number of people with family physicians, and to give access priority to vulnerable patients. As such, accessibility to a family physician will be assessed by two variables: all patients enrolled with a family physician through a GACO, and the number of vulnerable patients enrolled with a family physician through a GACO, that is, patients presenting one of the 19 vulnerability codes (*e.g.*, diabetes, mental illness, substance use) defined by the Quebec Insurance Board (RAMQ), which is the sole payer of physicians. These variables will be analyzed according to patients' characteristics: age, gender, source of referral to the GACO (such as the ER), priority code established by the intake nurse, as well as time elapsed before enrolment with a family physician.

### Data sources

We will use data from the GACOs' information system (SIGACO). This system is currently used by all GACOs in Quebec. We have privileged access to the SIGACO data through our primary partner, the Ministry of Health and Social Services (at no cost). The data are aggregated at the GACO level and anonymized. We will analyze all the data available since the year the GACOs were launched (2008) up to the most recent year for which data are available (2014). Note that we have access to the data in real time.

### Sample size

Nearly 600,000 patients have been enrolled with a family physician via one of the 93 GACOs between 2008 and today, according to the SIGACO system. There has been a clear progression over time (more than 100,000 enrolments in the past six months). These numbers are high enough to obtain the statistical significance required for the analyses to be carried out.

**Table 2:** **Details of the qualitative variables studied, their operationalization, and data sources**

Physician characteristics	CSSS characteristics	Local context
Sociodemographic profile (age, gender), practice profiles, type of primary care organization, proportion of time devoted to primary care, attitudes toward GACO, motivation for participation	Population focus vs. clientele focus	Integration of the population focus (CSSS leadership and coordination). Sociodemographic characteristics of the population (proportion of elderly persons, proportion of low-income persons/households, etc.), geographic characteristics of the territory (rural, urban, semi-urban)
	Leadership and governance: CSSS management team, role of the local family medicine department, organizational priorities	Territorial and interorganizational dynamics (collaboration among organizations)
	Resources: CSSS size, number of organizations making up the CSSS	Professional resources in the territory (number of family physicians, number of specialists), characteristics of the professionals in the territory (level of training, types of professionals), number of primary care organizations and prevalent organizational models
	Dynamics: Collaboration agreements with clinics in the territory, support for patient management	

#### Continuity

In this study, continuity is measured according to service utilization based on the frequency of primary care visits. This strategy will allow us to assess the `longitudinal' nature of the phenomenon, and is often used in primary care studies [62]. Continuity will be assessed on two dimensions: relational continuity and the continuity of the medical team.

Relational continuity is used to assess encounters between physicians and patients. It will be evaluated using two variables: patients' annual visits to the family physician with whom they were enrolled through the GACO, and attendance rates (usual provider of care) [63]. The variables will be analyzed over a two-year period beginning from the time when the patient was first enrolled with the family physician through the GACO. These variables are based on the literature, which shows that patients will prefer to consult the primary care physician who is following them. Medical team continuity will be used to assess visits to other physicians who are in the same primary care organization in which the patient's assigned physician practises.

These variables will be analyzed according to patients' characteristics: age, gender, and a comorbidity index that takes into account both the number of comorbid conditions and their severity. In Quebec, physicians are remunerated based on information provided for each patient visit: diagnostic codes and medical acts codes related to each visit are thus compiled, making it possible to determine whether the patient had a significant pathology.

### Data collection

To assess effects on continuity, we will us a RAMQ database containing all the billing codes for medical acts. Since a billing code is issued by the RAMQ for every patient referred through the GACOs, it will be easy to obtain the database covering all patient enrolled with a family physician through the GACOs.

### Sample size

The RAMQ database contains all patients enrolled with a family physician through the GACOs since their creation in 2008. These numbers are sufficiently high to obtain the statistical significance required for the analyses to be carried out. We will analyze all the data available from the year the GACOs were launched up to the year for which we have the most recent data from the RAMQ (2014).

#### Emergency room use

Emergency room utilization is often considered to be an inadequate substitute for primary care services, and as such, can be used to assess indirectly weaknesses in accessibility and continuity. Several studies seem to indicate that people with a family physician make less use of emergency room services [23],[27],[64]. Our aim is to assess the potential impact on emergency room use of people having become enrolled with a family physician through the GACOs. One of the variables used for this will be the average number of annual visits to the ER before and after the GACO enrolment, as well as the ratio of annual visits to the ER versus other primary care visits. As patients can see several physicians in one ER visit (RAMQ billing code), we will consider one visit to the ER per 24 hours [65]. Lastly, we intend to analyze the proportion of ER visits carried out with the family physician to whom the patient was enrolled through the GACOs. Our aim is to analyze whether, in certain contexts, particularly in rural areas, the ER is the setting used to see one's family physician [66]. This variable will be analyzed for exploratory purposes. All variables related to ER use will be analyzed according to the same patient characteristics as used for the continuity variables, *i.e.*, age and gender.

### Data collection

We will use the same RAMQ database for this component as for the continuity analysis.

### Analyses

First, we will prepare a profile of the effects on an annual basis for each GACO in Quebec. Then we will compare the annual effects among the GACOs to identify those with the best and worst outcomes. Finally, we will compare the GACO profiles over time and among themselves to analyze the impacts of policy changes on GACO outcomes, including changes to financial incentives for family physicians. For the effects on accessibility, the variables will be analyzed using linear regressions with repeated measures. For the effects on continuity, logistical or linear regressions will be performed depending on the nature of the data (continuous or categorical). Finally, for ER use, repeated measures ANOVA analysis will be performed. For each of the variables, sub-analyses will be performed according to the different patient characteristics (age, gender, principal diagnosis). We will use SAS 9.3 for all the analyses.

### Objective 2: Explain variations in the impacts by analyzing the characteristics of the GACOs and other factors influencing their implementation

Our aim is to understand better what factors explain the variations observed among the GACOs.

### Case selection

We will analyze four Quebec cases in depth. The cases will be selected to ensure we have contrasted cases in terms of effects. We intend to analyze two cases among those with the best outcomes on all the indicators analyzed in part one of the study (accessibility) and two cases at the other extreme for these indicators.

### Variables to be studied

We will characterize the GACOs under study by describing their visions and the resources, structures, and practices put in place. We will analyze four types of factors that have influenced the implementation of GACOs and the results obtained. Our analysis will look at: the characteristics of the physicians participating in the GACOs; the characteristics of patients on the GACO waiting lists and those of the patients enrolled with a family physician through GACOs; the characteristics of the CSSSs in which the GACOs have been implemented; and the characteristics of the local context. Table 2 presents the details of the variables to be studied, how they will be operationalized, and the data sources.

### Data sources

The primary sources of data will be the key actors involved and the grey literature. Our data collection strategy will involve individual semi-structured interviews. First, we will meet the key actors involved in the GACOs' operations (n = 5/case; total n = 20), including a nurse, the medical coordinator, a secretary, a manager, and the local head of the regional family medicine department. These interviews will enable us to better describe the characteristics of the GACOs (vision, structures, resources, tools) and the different factors influencing their implementation (family physicians, patients, CSSS, local context). Second, we will interview physicians registered with GACOs (n = 4/case; total n = 16). The physicians will be identified with the help of the local medical coordinator. These interviews will provide a better understanding of the factors that facilitate and impede physicians' participation in GACOs. All interviews will be audio-recorded and subsequently transcribed verbatim by a professional transcriptionist.

### Analysis

The research technician will code every interview. The data will be coded using NVivo (QSR) software, such that, in each transcript, the different points addressed in the interviews will be labelled. We have established a summary list of initial codes based on our conceptual model (Table 2). This list shows the different characteristics of the GACO models that have been implemented and the factors that might influence them. This list will be modified and enhanced over the course of the analyses. Coding will be controlled using a double-coding technique. Coding will be done primarily by the research technician and one researcher (Breton), who will code the first interviews in parallel and independently and then will compare their results. Where there are differences, they will clarify these, refine the codes, and repeat the coding. This process will be repeated until we obtain inter-coder agreement of more than 90% [54].

We will start with an intra-case analysis. We will analyze the coded material and the effects variables and will synthesize the results using tables and matrices [54]. These will summarize the data obtained for each case being studied. The matrices will present the results by grouping together the codes according to the different themes proposed in our conceptual framework and any new themes that might emerge during the analysis. From these tables and matrices, for each case studied, we will prepare a `thematic network analysis,' which consists of identifying the relationships among the different dominant themes (organizing themes) and the characteristics that make them up (basic themes) using graphic representations [67]. We will use this analytical approach to gain a better understanding of the relationships among the different factors and the observed effects. We will then use this material to perform a cross-sectional inter-case analysis. We will analyze the similarities and differences among the four case studies, as illustrated by the thematic network analysis, and will draw out the key lessons. This analysis strategy will help us in developing a set of recommendations regarding GACO models that perform well and less well (in relation to outcomes), their key components, and the environmental factors that facilitate or impede implementation. We will prepare a synthesis report on the key lessons that can facilitate better implementation of GACOs in different implementation contexts.

The study has been approved by the Research Ethics Board of the Centre hospitalier universitaire de Sherbrooke (ref. number CHUS14-091).

### Study validity

#### Construct validity

This is ensured by a detailed conceptual model that identifies the different variables to be studied [68]. These variables are based on an exhaustive literature review and on results obtained and observations made during the monitoring project.

### Internal validity

This will be ensured by using an analytical framework, doing systematic coding, and rigorously organizing the data, as well as by triangulating the qualitative data through analysis of different perspectives [69]. Analysis of contrasting cases will also reinforce internal validity. For the quantitative component, a temporal bias could affect the results, as patients' health status could change over time. However, several indicators deal with proportions of numbers of visits. Also, the same patients are followed over time, which will make it possible to control for patient characteristics.

### External validity

Using multiple case studies with contrasted representations of implementation contexts will reinforce external validity [68]. It will also be reinforced by holding discussion sessions between the research team and the pan-Canadian committee to better understand the factors producing the effects in the different implementation contexts.

### Fidelity

The detailed descriptions at each stage of our multiple case study will ensure fidelity, especially given that the procedure followed for each activity will be described and presented in a research report.

## Discussion

To our knowledge, no research has yet been done on the implementation and effects of these new and complex organizational models, apart from one exploratory study we conducted in one region of Quebec [45]. The study we propose will help to fill in these gaps and respond to these questions. This study will enable us to learn about the mechanisms by which the effects are produced, including several aspects related to the implementation of waiting lists: the phenomenon of registering and selecting clienteles, the roles of professionals in the processing of requests, the influence of incentives for physicians' participation in waiting lists. This project will contribute not only to the body of scientific knowledge on the organization of primary care innovations, but will also be very useful to decision-makers and clinicians in the different Canadian provinces by providing a better understanding of the issues and promising strategies for implementing waiting lists in Quebec and elsewhere in Canada.

## Authors' contributions

MB led the coordination and the conceptualization of the study. MB, ABrousselle and DR wrote the first draft, ABoivin, CL, NT, CAD, KN and DB critically reviewed it and provided comments to improve the manuscript. All authors have read and commented on the final manuscript.

## Authors’ original submitted files for images

Below are the links to the authors’ original submitted files for images. Authors’ original file for figure 1

## Abbreviations

MSSS: Ministry of Health and Social Services
CSSS: Health and social services centre
RAMQ: Quebec Health Insurance Board
GACO: Guichet d'accès aux clienteles orphelines (waiting list for `orphan' clienteles)
