# Supplementary figures and images for: Evaluation of the implementation of centralized waiting lists for patients without a family physician and their effects across the province of Quebec
Source: Implement Sci. 2014 Sep 4;9:117. doi: 10.1186/s13012-014-0117-9 (PMC4159553; doi:10.1186/s13012-014-0117-9)

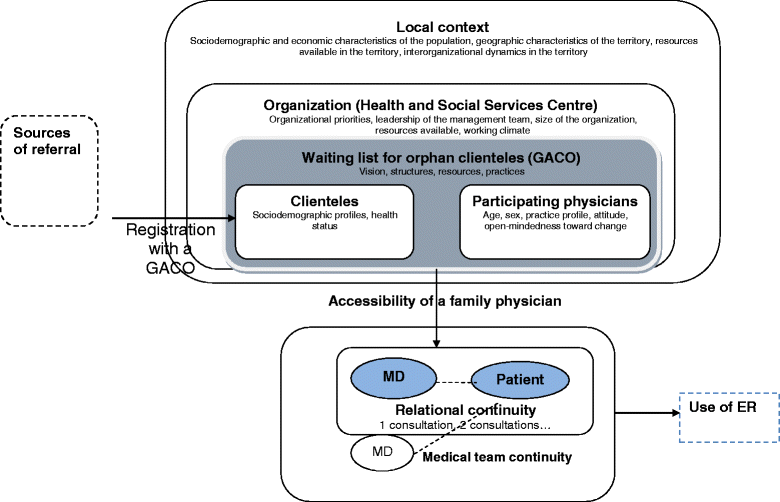

Supplement: Supplementary file 1 — Authors’ original file for figure 1 [file 13012_2014_117_MOESM1_ESM.gif]
